# Projections from the dorsal and ventral cochlear nuclei to the medial geniculate body

**DOI:** 10.3389/fnana.2014.00010

**Published:** 2014-03-05

**Authors:** Brett R. Schofield, Susan D. Motts, Jeffrey G. Mellott, Nichole L. Foster

**Affiliations:** ^1^Department of Anatomy and Neurobiology, Northeast Ohio Medical UniversityRootstown, OH, USA; ^2^School of Biomedical Sciences, Kent State UniversityKent, OH, USA; ^3^Department of Physical Therapy, Arkansas State UniversityJonesboro, AR, USA

**Keywords:** thalamus, multipolar cells, T-stellate, magnocellular pathway, fear conditioning, lemniscal pathway, multimodal processing, collateral projections

## Abstract

Direct projections from the cochlear nucleus (CN) to the medial geniculate body (MG) mediate a high-speed transfer of acoustic information to the auditory thalamus. Anderson etal. (2006) used anterograde tracers to label the projection from the dorsal CN (DCN) to the MG in guinea pigs. We examined this pathway with retrograde tracers. The results confirm a pathway from the DCN, originating primarily from the deep layers. Labeled cells included a few giant cells and a larger number of small cells of unknown type. Many more labeled cells were present in the ventral CN (VCN). These cells, identifiable as multipolar (stellate) or small cells, were found throughout much of the VCN. Most of the labeled cells were located contralateral to the injection site. The CN to MG pathway bypasses the inferior colliculus (IC), where most ascending auditory information is processed. Anderson etal. (2006) hypothesized that CN-MG axons are collaterals of axons that reach the IC. We tested this hypothesis by injecting different fluorescent tracers into the MG and IC and examining the CN for double-labeled cells. After injections on the same side of the brain, double-labeled cells were found in the contralateral VCN and DCN. Most double-labeled cells were in the VCN, where they accounted for up to 37% of the cells labeled by the MG injection. We conclude that projections from the CN to the MG originate from the VCN and, less so, from the DCN. A significant proportion of the cells send a collateral projection to the IC. Presumably, the collateral projections send the same information to both the MG and the IC. The results suggest that T-stellate cells of the VCN are a major source of direct projections to the auditory thalamus.

## INTRODUCTION

The first experimental evidence for a direct projection from the cochlear nucleus (CN) to the medial geniculate body (MG) was published over 70 years ago, when Woollard and Harpman (1940) described a few degenerating axons entering the MG after lesioning the CN in a guinea pig. This result, from a single animal, was not the focus of the paper and was largely ignored for many years. In the early 1970s, Strominger and Strominger (1971) and Strominger (1973), described degenerating fibers in the MG of rhesus monkeys after lesions of the CN, but concluded that the number of axons was negligible. Strominger et al. (1977) described the patterns of degeneration following lesion of the CN in a single chimpanzee. The report focused on this projection, and the authors argued strongly for the existence of a direct projection from the CN to the magnocellular and principal divisions of the MG. More recently, investigators have taken advantage of sensitive anatomical tracers to demonstrate a similar pathway in rats (Malmierca et al., 2002) and guinea pigs (Anderson et al., 2006). The latter study also described acoustically driven responses from neurons in the MG. The results showed short-latency responses in the MG, with a significant proportion of cells responding to a click stimulus in less than 6.5 ms. These short latencies were present only in the medial division of the MG, where the CN to MG pathway appears to terminate. Taken together, the anatomical and physiological results suggest the existence of a high-speed pathway from the CN to the MG that bypasses the inferior colliculus (IC), where the majority of ascending auditory information is processed.

The studies cited above demonstrate a pathway from the CN to the MG in guinea pigs, rats, chimpanzees, and perhaps, rhesus monkeys, suggesting that such a pathway is present in a broad range of species. However, a number of questions remain regarding both the origins and terminations of such a pathway. Do the projections arise from both the dorsal and ventral CN and, if so, what are the relative contributions of the two nuclei? If there are projections from the ventral CN, from what cell types do they arise? Is there a projection to the ipsilateral MG? The present series of experiments addresses these questions. In addition, we have tested a suggestion by Anderson et al. (2006), who noted that the morphology of CN axons that terminate in the MG is similar to that of CN axons that terminate in the IC. They suggested that the axons may arise as collateral branches from the same population of cells in the CN.

## MATERIALS AND METHODS

Experiments were performed on 23 adult guinea pigs of either sex (one albino animal obtained from Charles River Laboratories, Wilmington, MA, USA; 20 pigmented animals obtained from Elm Hill Breeding Labs, Chelmsford, MA, USA, and two pigmented animals bred from stock obtained from the Elm Hill colony). All procedures were approved by the Institutional Animal Care and Use Committee and administered following the National Institutes of Health guidelines for the care and use of laboratory animals. Efforts were made to minimize suffering and the number of animals used.

### SURGERY AND PERFUSION

Prior to surgery, each guinea pig was anesthetized with halothane (3.5% for induction, 2.0–2.75% for maintenance) in oxygen and nitrous oxide or with isoflurane (4–5% for induction, 1.75–3% for maintenance) in oxygen. The animal was given atropine sulfate (0.08 mg/kg, i.m.) to reduce bronchial secretions. The eyes were kept moist with a coating of Neosporin Ophthalmic ointment or Moisture Eyes PM moisturizer. The animal was placed in a stereotaxic frame on a feedback-controlled heating pad to maintain body temperature. For surgery, an incision was made in the scalp and the margins of the incision were injected with a long-lasting local anesthetic (0.25% bupivacaine; Sensorcaine; Astra USA, Inc., Westborough, MA, USA). Injections were made according to stereotaxic coordinates. The animal was placed in the frame with the incisor bar positioned 5.0 mm below earbar zero. The range of coordinates for MG injections was: 4.6–5.4 mm rostral to earbar zero; 4.0–4.8 mm lateral to midline; 4.2–4.8 mm dorsal to earbar zero. A dental drill was used to open the skull at appropriate locations.

Five different fluorescent retrograde tracers were used: Fast Blue (FB, 5% aqueous solution; EMS-Chemie GmbH, Gross-Umstadt, Germany), red or green fluorescent microspheres (“RetroBeads,” both from Lumafluor, Naples, FL, USA) and FluoroGold (FG, 4% in water; FluoroChrome, Inc., Englewood, CO, USA); fluorescein dextran (FD; 10% in saline; 10 k molecular weight; Invitrogen). Large injections were made with a 1 μl Hamilton microsyringe. Small injections were made with a micropipette (25–35 μm inside diameter) attached to a Nanoliter Injector (World Precision Instruments, Sarasota, FL, USA). Volumes of 13.8–69.0 nl were injected at a single site by injecting small amounts (13.8 nl) at 1 min intervals until the desired total was reached.

Tracers were injected into 29 MGs in 21 animals (**Table 1**). For control purposes, tracers were injected into the nucleus of the brachium of the IC (NBIC) in two animals (**Table 1**). Tracers were also injected into the IC in 14 of the animals that had injections into one or both MGs (**Table 1**). In one of these cases, the green bead liquid was mixed with an equal volume of fluorescein dextran (FD, 10% solution in saline; 10 k molecular weight, Invitrogen). Each tracer was injected at multiple sites (2–6) in one IC. Each site received an injection of 0.1–0.2 μl. Each tracer was injected with a microsyringe used only for that tracer. Following the injections, the exposed brain was covered with Gelfoam and the scalp was sutured. Ketoprofen (3 mg/kg, i.p.) was injected to provide post-operative analgesia. After surgery the animal was placed in a clean cage and monitored until it regained the ability to stand. At that point, it was transported in its cage to the animal facility.

**Table 1:** Injection locations.

Case	Left MG*	Right MG*	Left IC	Right IC	Right NBIC
GP482	FG	GB	RB		
GP484	FG	GB			
GP500	FG		GB	RB	
GP501	FG		GB	RB	
GP579	FG	RB			
GP585	GB	RB	FB		
GP586	GB	RB	FB		
GP587	GB	RB	FB		
GP591	FG		FD/GB		
GP595	GB	RB	FG		
GP597	FG		GB		
GP604	FG		GB		
GP605	FG		GB		
GP608		FG		GB	
GP609	FB		GB		
GP610	FB		GB		
GP689	RB (MGm, MGsg)	GB (MGm)			
GP693	RB (MGd)				
GP695	RB (MGv)				
GP696		FG			
GP698	RB (MGsg)				
GP702					RB
GP712					GB

Table showing the tracer injected into the medial geniculate body (MG), inferior colliculus (IC), or nucleus of the brachium of the IC (NBIC). *Injections into the MG included all subdivisions unless otherwise noted. In select cases, small injections were made into one or two subdivisions, indicated in parentheses. In some cases, a different tracer was injected into another brain region for a different experiment; data from these injections are not reported here. FB, fast blue; FD/GB, mixture of fluorescein dextran and green beads; FG, FluoroGold; GB, green beads; RB, red beads; MGd, dorsal MG; MGm, medial MG; MGsg, suprageniculate MG; MGv, ventral MG.

After 4–25 days, the animal was sacrificed with an overdose of pentobarbital (440 mg/kg; i.p.) or isoflurane (inhalation of 5% isoflurane in oxygen; Note: the longer survival times were used in some cases to ensure that sufficient time was allowed for retrograde transport and, in other cases, to enhance the visualization of cortical axons labeled by injection of anterograde fluorescent tracer into the auditory cortex as part of other experiments. In these latter cases, the cortical axons were labeled with a red tracer, tetramethylrhodamine dextran, and the tracers injected into the MG were viewed in separate, i.e., non-red, fluorescent filters so that MG-projecting cells could be identified unambiguously). Once breathing had stopped and the animal showed no withdrawal reflex to a paw pinch or touch of the cornea, the animal was perfused through the aorta with approximately 100 ml of Tyrode’s solution (pH 7.4), 350 ml of 4% paraformaldehyde in 0.1 M phosphate buffer, pH 7.4 (PB), and 350 ml of 4% paraformaldehyde with 10% sucrose in PB. The brain was then removed and stored at 4°C in 4% paraformaldehyde with 25 or 30% sucrose in PB. The following day, the brain was cut on a sliding microtome into 40 or 50 μm thick transverse sections. Sections were collected in six series. At least four series of sections were mounted on gelatin-coated slides and allowed to dry. In most cases with small injections, sections through the thalamus were stained for cytochrome oxidase activity to identify MG subdivisions (Anderson et al., 2007). Stained sections were mounted on slides and dried overnight. All sections were then coverslipped with DPX (Aldrich Chemical Company, Inc., Milwaukee, WI, USA).

### DATA ANALYSIS

Subdivisions of the MG were identified as described in Anderson et al. (2007). For analysis of injections into the IC, the IC subdivisions were identified by comparison with sections stained with thionin and/or nicotinamide adenine dinucleotide phosphate diaphorase (NADPH) or anti-neuronal nitric oxide synthase and criteria described previously (Faye-Lund and Osen, 1985; Dawson et al., 1991; Druga and Syka, 1993, 2001; Malmierca et al., 1995; Paloff and Hinova-Palova, 1998; Coote and Rees, 2008).

Retrogradely labeled cells in the CN were examined with 20× or 40× objectives and plotted in a series of sections (spaced 300 μm apart) through the CN using a Neurolucida plotting system (MBF Bioscience, Williston, VT, USA) connected to a Zeiss Axioplan II fluorescence microscope. The borders of nuclei were identified with adjacent thionin-stained sections or by removing the coverslips from the plotted sections, re-hydrating them, staining them with thionin, and re-applying coverslips. Plots were exported to Adobe Illustrator for final illustration.

For analysis of cell morphology, selected cells were drawn with a 40× objective and a Zeiss camera lucida attached to a Zeiss Axioskop fluorescence microscope. The locations of the cell bodies were indicated on low-magnification drawings made with a 2.5× objective. The drawings were inked, scanned into Adobe Photoshop using a flatbed scanner (Canon LiDE 60) and transferred to Adobe Illustrator for labeling.

Photomicrographs were taken with the Zeiss Axioskop microscope and an attached Optronics Magnafire camera or with a Zeiss AxioImager Z1 microscope equipped with Axiocam HRc and HRm cameras. For the latter cases, initial image processing (e.g., contrast adjustment) was done with Axiovision software (Zeiss, Axiovision Version 4.6). For all images, Adobe Photoshop was used to make final adjustments of intensity levels and color balance, to arrange and label photographs, and to erase background outside the sections.

### QUANTIFICATION OF LABELED CELLS

Preliminary observations indicated that labeled cells in the CN were more numerous on the contralateral side and, on both sides, were more numerous in the VCN than in the DCN. To quantify these distributions, every labeled cell was counted in 1–3 series of sections evenly spaced through the entire CN. The relative numbers of labeled cells were then expressed as percentages.

For analysis of collateral projections, labeled cells in the CN were examined for the presence of two or more tracers after injection of different tracers in multiple targets. Possible projection patterns that were assessed included projections to (1) ipsilateral and contralateral MG; (2) one IC and one MG on the same side of the brain; (3) one IC and one MG on opposite sides of the brain; (4) both MGs and one IC; (5) one MG and both ICs. For quantification, all CN cells that contained tracer from either MG were counted in a series of every sixth section through the entire CN. The only pattern of collateral projections that was seen with any regularity was a projection to the contralateral MG and the contralateral IC. The relative contribution of the collateral projection compared to the overall projection to the MG was then expressed according to (# double-labeled cells)/(# of cells labeled from the contralateral MG).

## RESULTS

### RETROGRADE LABEL FOLLOWING MG INJECTIONS

**FIGURE 1:**
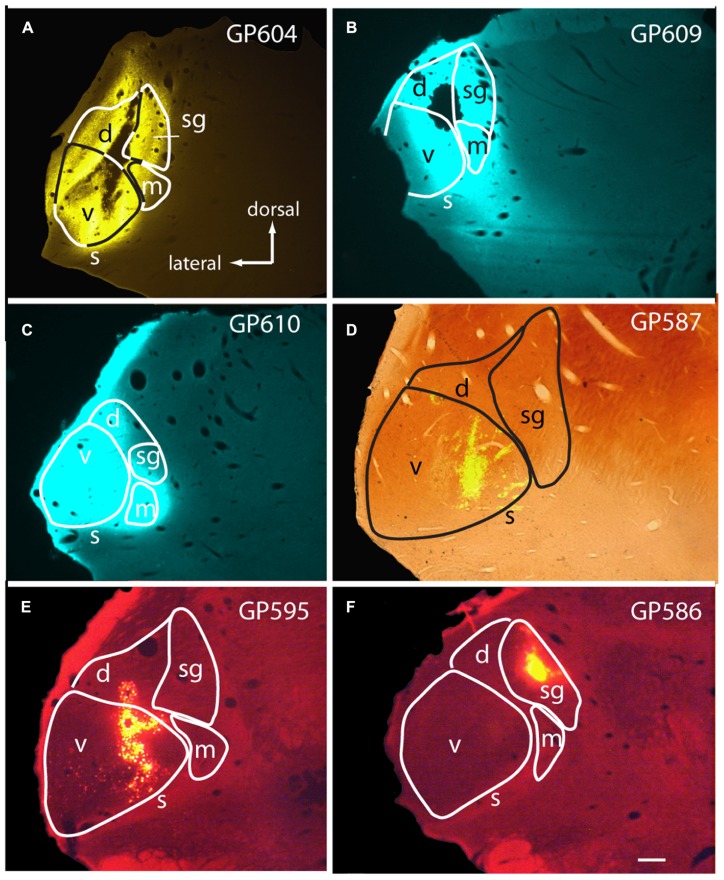
**Photomicrographs showing representative injection sites in the medial geniculate body (MG). (A–C)** Large injections of FluoroGold **(A)** or Fast Blue **(B,C)**. **(D)** Small injection of green beads, confined to the ventral subdivision of the MG, visualized in a section that was also stained for cytochrome oxidase activity (combined fluorescence and brightfield image). **(E,F)** Injections of red beads into the ventral and dorsal MG **(E)** or suprageniculate subdivision **(F)**. Experiment numbers (e.g., GP604) are shown for each panel. Transverse sections; scale bar = 0.5 mm. d, dorsal MG; m, medial MG; s, shell of MG; sg, suprageniculate MG; v, ventral MG.

Large injections encompassed much of the MG as well as parts of the surrounding nuclei, most often dorsal to the MG (**Figures 1A–C**). The smallest injections were confined to a single subdivision (e.g., **Figures 1D,F**) or, in some cases, encroached on a second subdivision of the MG (**Figure 1E**; center of injection in ventral MG, with some tracer also in dorsal MG). The caudal-most injections may have encroached on the NBIC. None of the injections spread to the pretectal nuclei or to the superior colliculus.

**FIGURE 2:**
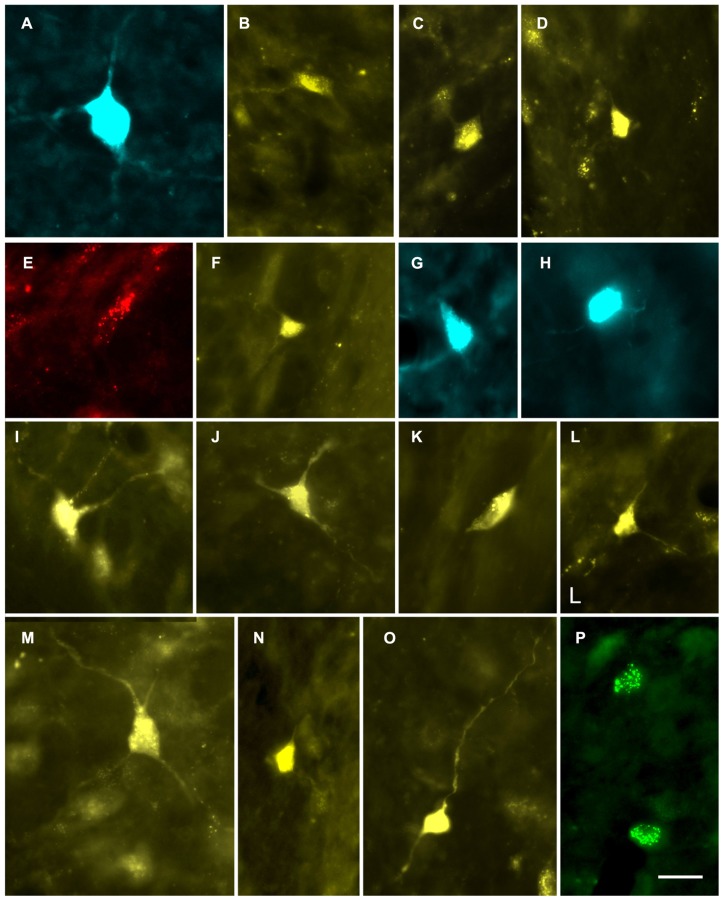
**Photomicrographs showing labeled cells in the CN. (A,B)** Cells in the deep layer of the dorsal cochlear nucleus that project to the contralateral MG. **(A)** Fast Blue (FB)-labeled giant cell. GP609. **(B)** FluoroGold (FG)-labeled cell. GP500. **(C,D)** FG-labeled cells in the VCN that project to the *ipsilateral* MG. GP591 **(C)**; GP608 **(D)**. **(E–P)** Labeled cells in the VCN that project to the *contralateral* MG. Cells were labeled with red beads **(E)**, Fast Blue **(G, H)**, FluoroGold **(F, I–O)** or green beads **(P)**. GP586 **(E)**; GP604 **(F)**; GP610 **(G,H)**; GP591 **(I–O)**; GP587 **(P)**. Scale bar = 20 μm (applies to all panels). Dorsal is up; lateral is to the right except for panels **C** and **D**, where lateral is to the left.

**FIGURE 3:**
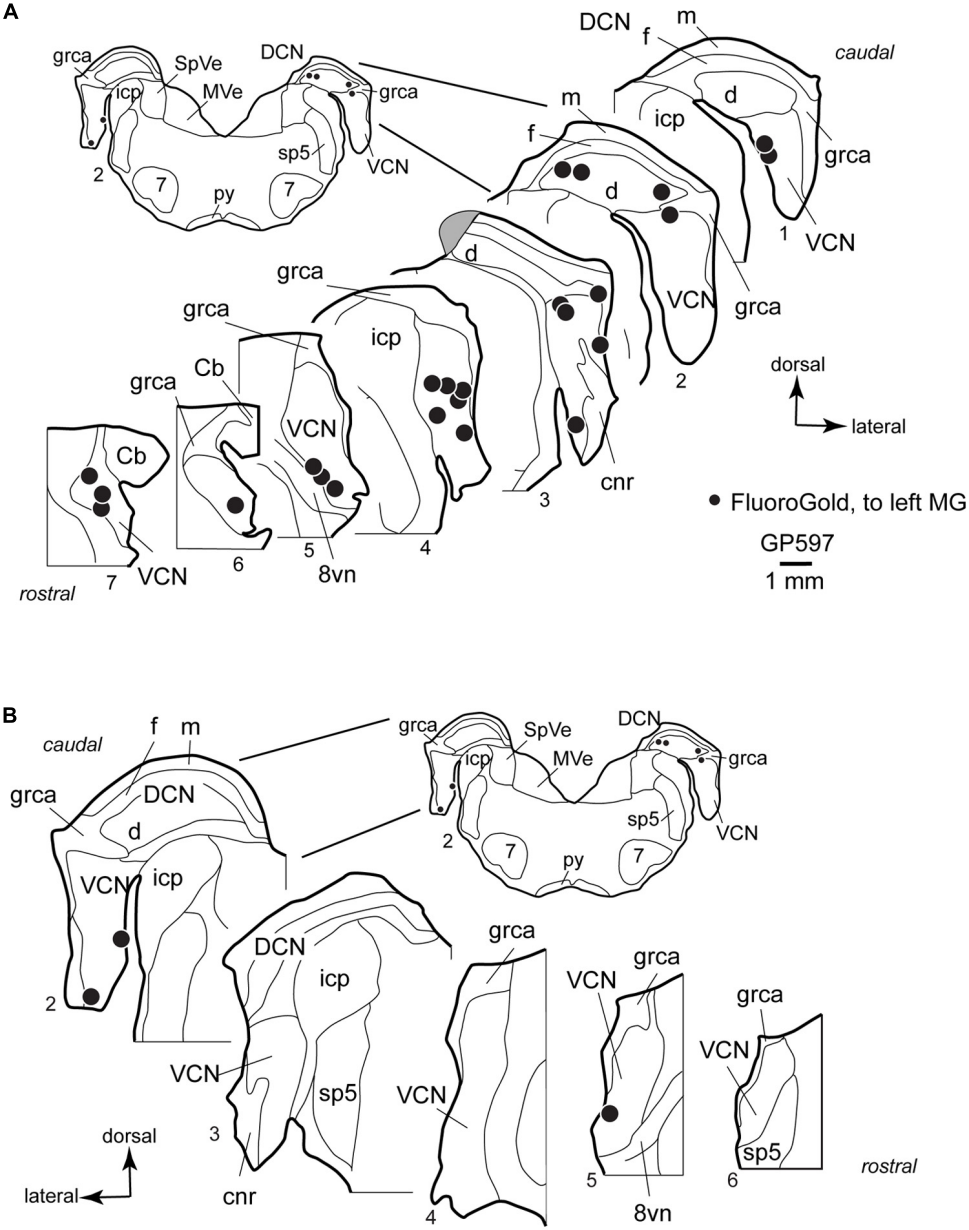
**Plots showing the distribution of labeled cells in the cochlear nucleus following a large injection of FluoroGold into the left medial geniculate body.** Enlargements of the cochlear nucleus are shown, with the inset providing orientation. Plots are contralateral **(A)** or ipsilateral **(B)** to the injection. A photograph of the injection site for this case is presented below (**Figure 6C**). Each black dot represents one or more labeled cells. Sections are numbered from caudal to rostral and are spaced 300 μm apart. See list for abbreviations. Gray area in **A**, Section 3, indicates tissue damaged during processing.

Injection of tracers into the MG labeled cells in the CN bilaterally (**Figure 2**). The results were similar qualitatively with all tracers and with all survival times tested. The results from the one albino animal were also similar to those from the pigmented animals. **Figure 3** shows the distribution of cells in the CN after a large injection of FG into the left MG. The majority of labeled cells were located on the contralateral side (average: 90%; range 76–100%; eight experiments). On the contralateral side, most of the labeled CN cells (average: 87%; range 62–100%; nine experiments) were located in the VCN.

In the VCN, the cells were distributed through most of the rostral-caudal extent, and could be found in all areas except the octopus cell area and granule cell area. Labeled cells were often located near the borders of the VCN, and occupied the small cell cap in some instances (cf. Hackney et al., 1990). The small cell cap abuts the granule cell lamina separating the VCN and DCN. Small cells are also found along the medial and lateral margins of the VCN. The borders between the areas with small cells, including the cap area, and the more centrally located “magnocellular” part of the VCN are difficult to discern in guinea pigs. However, the spread of labeled cells across the full width of the VCN demonstrates clearly that some of the cells labeled in the present study were located in the magnocellular parts of the VCN (e.g., **Figure 3A**, section 4). A smaller number of labeled cells were present in the DCN, where they were concentrated in the deep layer. Though not present in the sections illustrated, there were rare occurrences of labeled cells in the fusiform cell layer and in the molecular layer. On the side ipsilateral to the injection, a small number of labeled cells were present in the VCN (**Figure 3B**). As on the contralateral side, the cells in the ipsilateral VCN were often near the edges of the nucleus. The case illustrated had no labeled cells in the DCN on the ipsilateral side; a few such cells were observed in the deep DCN in other cases.

In general, the number of cells labeled by an injection reflected the extent to which the injection site included the medial subdivision of the MG. Nonetheless, even very small injections of beads confined to one subdivision labeled cells in the CN; these were contralateral to the injection. **Figure 2E** shows a cell in the VCN labeled by an injection of red beads in the suprageniculate subdivision (injection site shown in **Figure 1F**). **Figure 2P** shows two VCN cells labeled by an injection of green beads into the ventral subdivision (injection site shown in **Figure 1D**). A small injection into the MGd also labeled cells in the contralateral VCN (not shown). In all cases in which the injection site was outside the MGm, the number of labeled CN cells was noticeably smaller than after injections that included the MGm.

**FIGURE 4:**
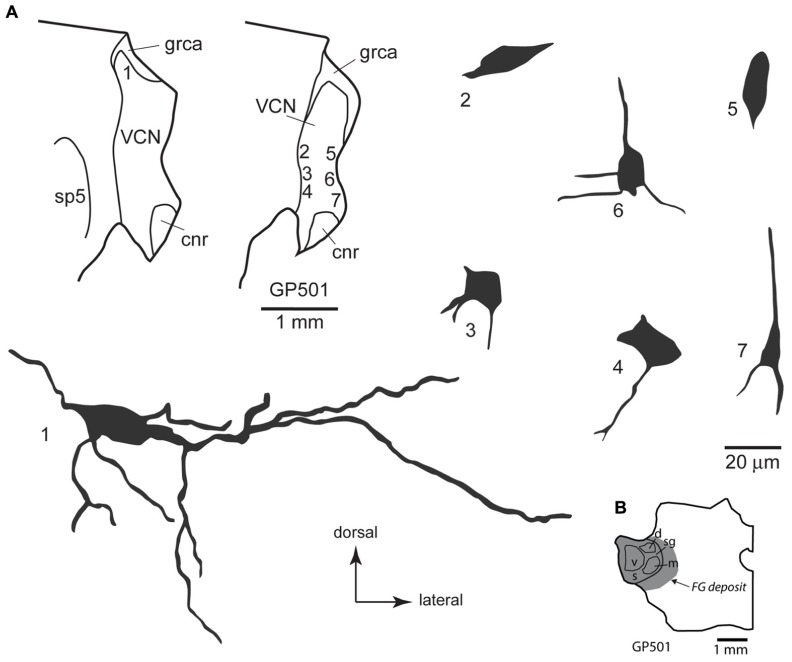
**(A)** Camera lucida drawings of cells in the ventral cochlear nucleus labeled by injection of FluoroGold in the contralateral medial geniculate body. Dendritic labeling was usually very limited, but somatic morphology frequently suggested multiple dendrites. Some cells (e.g., cell #1) appear to be members of the multipolar class, whereas the small size of other somas (e.g., cell #7) suggest that members of the small cell class were also labeled. The cells were drawn from two sections, as shown in the inset. Numbers next to each cell correspond to their location as shown in the inset. Transverse sections. **(B)** Drawing of a transverse section through the left thalamus showing the spread of a FluoroGold deposit across all subdivisions of the medial geniculate nucleus in the animal from which the cells in part A were drawn. cnr, cochlear nerve root; d, m, s, sg, v, dorsal, medial, shell, suprageniculate and ventral subdivisions of the MG; FG, FluoroGold; grca, granule cell area; sp5, spinal trigeminal tract; VCN, ventral cochlear nucleus.

**FIGURE 5:**
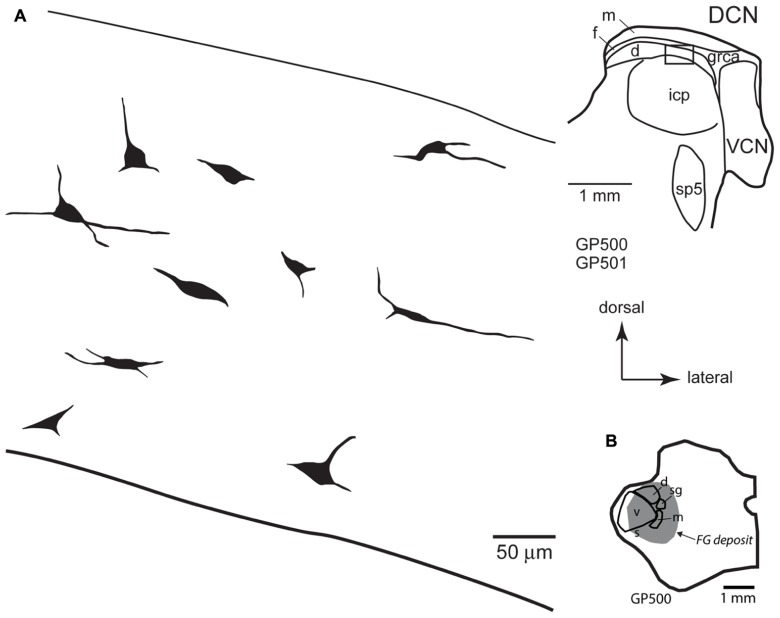
**(A)** Camera lucida drawings of cells in the deep layer of the dorsal cochlear nucleus labeled by injection of FluoroGold in the contralateral medial geniculate body. The cells were drawn from numerous sections from two different animals (GP500, GP501); the drawings were collected into a single composite drawing, preserving their relative orientation and depth with respect to the laminar borders. Inset: representative drawing of the DCN, with a box indicating the approximate location of the enlarged drawings of labeled cells. Transverse sections. **(B) **Drawing of a transverse section through the left thalamus showing the spread of a FluoroGold deposit across all subdivisions of the medial geniculate nucleus in the animal GP500. The cells drawn in panel **A** were obtained from this injection and the one shown in **Figure 4B**. d, m, s, sg, v, dorsal, medial, shell, suprageniculate, and ventral subdivisions of the MG; FG, FluoroGold; DCN, dorsal cochlear nucleus; d, f, m, deep, fusiform cell, and molecular layers of the DCN; grca, granule cell area; icp, inferior cerebellar peduncle; sp5, spinal trigeminal tract; VCN, ventral cochlear nucleus.

Some of the retrogradely labeled cells were filled sufficiently to identify their morphological class. Identifiable cells in the VCN, contralateral or ipsilateral to the injection in the MG, belonged to the multipolar and small cell classes (**Figure 4**). We found no evidence for labeled globular bushy cells, spherical bushy cells or octopus cells, although we cannot rule out the presence of a few such cells among those that could not be classified. In the DCN, most of the cells were in the deep layer, but few were labeled sufficiently for classification. Many of these cells on both sides of the brain had an elongated cell body oriented parallel to the layers (**Figure 5**). The rare exceptions included a couple examples of giant cells (e.g., **Figure 2A**) and, in one case, a fusiform cell (not shown; located contralateral to the injection). The latter cell was labeled by an injection that encroached on the NBIC, so we cannot rule out the possibility that this nucleus was the target of the fusiform cell axon (discussed below).

### CONTROL INJECTIONS

In two cases, we injected red or green beads into the NBIC, which is located just caudal to the MG and which receives direct projections from the CN (unpublished observations). In these cases, we observed labeled cells in the VCN bilaterally. These cells were few in number, but were morphologically indistinguishable from those labeled by the injections into the MG. In one of the cases, there were labeled cells in the DCN on the contralateral side but not on the ipsilateral side. The DCN cells were located in the deep layer and were similar morphologically to the small cells labeled after MG injections. No fusiform cells were labeled in these cases.

### DOUBLE-LABEL FOLLOWING INJECTIONS IN THE MG AND THE IC

**FIGURE 6:**
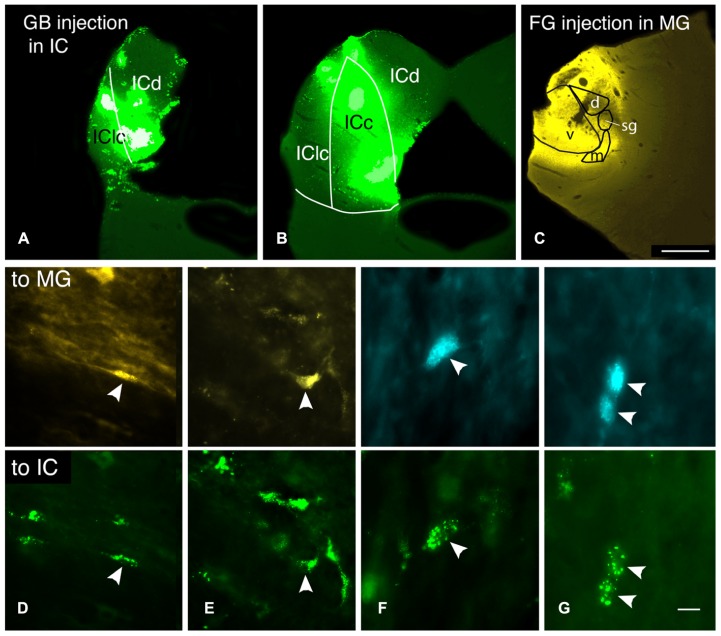
**Photomicrographs showing injection sites and double-labeled cells after injections of different tracers into the contralateral medial geniculate body (MG) and the contralateral inferior colliculus (IC). (A–C)** Transverse sections showing representative injections sites in the IC **(A,B)** and the MG in case GP597.** (A,B)** Deposits of green beads in two sections through the IC, one located very caudally **(A)** and one in the rostral third of the IC **(B)**. IC subdivisions are shown, illustrating the spread of green beads into the lateral cortex (IClc), the dorsal cortex (ICd) and the central nucleus (ICc). **(C) **FluoroGold deposit in the MG. Scale bar = 1 mm and applies to **A–C**. **(D–G)** Photomicrographs showing examples of double-labeled cells (arrowheads). Each column shows a single field of view, showing one or more cells labeled with the tracer injected into the MG (top panel) or into the IC (bottom panel). **(D)** Cell in the dorsal cochlear nucleus labeled with FluoroGold and green beads. Additional cells labeled only with FluoroGold or green beads are also visible. GP597. **(E)** Cell in the ventral cochlear nucleus labeled with FluoroGold and green beads. Additional cells are single-labeled only with green beads. GP597. **(F,G)** Cells in the ventral cochlear nucleus labeled with Fast Blue (top) and green beads (bottom). GP610. Scale bar in G = 20 μm **(D,E)**; 10 μm **(F,G)**. All panels: transverse sections, dorsal is up; for **A–C**, lateral is to the left; for **D–G**, lateral is to the right.

Each animal received an injection of a fluorescent tracer into one MG and another tracer into the opposite MG and/or one or both ICs; some animals received injections of three distinguishable tracers (**Table 1**; **Figure 6**). The combined injections allowed us to identify single cells that contained more than one tracer and thus sent axon branches to multiple targets. We observed a substantial number of double-labeled cells that sent axon branches to the contralateral MG and the contralateral IC. The double-labeled cells were observed in numerous cases with various tracer combinations. We chose seven cases with the most labeled cells for quantitative analysis. In these cases, 14–37% of the labeled cells in the VCN were double-labeled (average: 24%; 167 cells). The DCN had fewer labeled cells (as expected from the single-label data described above); nonetheless, 3 of the 30 labeled DCN cells were double-labeled (based on six cases; the DCN was damaged in one of the cases used for VCN analysis and thus was excluded from quantitative analysis for DCN label).

Our combination of injection sites would have allowed us to identify several additional axonal branching patterns, including cells that project to both left and right MG or that project to an IC and an MG on different sides of the brain (**Table 1**). We observed two CN cells that were labeled by injections into the left and right MG and one cell that was labeled by injection in the contralateral MG and the ipsilateral IC. Such cells were quite rare, suggesting that these branching projection patterns are also rare. We never observed triple-labeled cells.

## DISCUSSION

The results confirm the existence of a projection from the CN to the MG in guinea pigs. The projection is bilateral with a contralateral dominance, and terminates primarily in the MGm. Two new findings are noteworthy. First, the majority of cells that contribute to this pathway are in the VCN. This finding has implications for interpreting the results of anterograde tracer deposits placed in the DCN (Anderson et al., 2006). This result also differs from a report in rats, where the DCN was considered the main source of projections. The second major finding is that many of the CN cells that project to the MG send collateral projections to the IC. This result has implications for the identity of the projecting cells (likely to be T-stellate cells and small cells) as well as the functions of the CN to MG pathway.

### COMPARISON WITH PREVIOUS LITERATURE

Woollard and Harpman (1940) described degenerating fibers in the MG of a guinea pig after lesion of one CN, but they did not provide details of the origins or termination patterns. Strominger and Strominger (1971), Strominger (1973), and Strominger et al. (1977) concluded that a direct projection from CN to MG is minimal in rhesus monkeys but present in “higher primates” like chimps, where the pathway terminates in the MGm and in the “principal division” (=main target of IC projections; Strominger et al., 1977). Much more recently, Malmierca et al. (2002) demonstrated a direct CN to MG pathway in rats. Most of their data were based on anterograde transport from tracer deposits into the DCN. They concluded that the pathway terminates primarily in contralateral MGm. They also identified the cells of origin based on deposits of retrograde tracer into the MGm in two animals. They observed a number of presumptive fusiform (a.k.a. pyramidal) cells and a larger number of giant cells in the deep layer of the DCN. They also observed small cells in the VCN, both around the margins of the posterior VCN and in the small cell cap. These earlier studies were based largely (Malmierca et al., 2002) or exclusively (other studies cited above) on anterograde transport or axonal degeneration. The present study, based on a large number of experiments with retrograde tracers, confirms many of the earlier findings and extends the information on the distribution and identity of the cells of origin. We also present the first data on collateral projections, whereby many CN-MG cells send branching axonal projections to the IC as well as the MG.

The major difference from earlier studies concerns the relative contributions of the DCN and the VCN to CN-MG projections. Malmierca et al. (2002) concluded that the DCN contains the majority of CN-MG cells in rats, whereas we found that the VCN contains a large majority (on average, 87%) of CN-MG cells in guinea pigs. The discrepancy could reflect a difference between species or there could be differences based on technical issues. The results from rats were based on six cases, two of which labeled cells in the CN (Malmierca et al., 2002). These two cases used horseradish peroxidase as a tracer, raising the possibility that the different results could reflect the tracers used. It is well established that different tracers have different properties and limitations (e.g., Aschoff and Holländer, 1982; Köbbert et al., 2000; Burger et al., 2005; Schofield et al., 2007; Schofield, 2008), so the robustness of any conclusion is enhanced by consistency of results across tracers. The present results are based on four different retrograde tracers injected into 29 different MGs. The results were consistent across all cases. Recent results, obtained with anterograde tracers, provide additional information relevant to this issue.

Anderson et al. (2006) injected the anterograde tracer biotinylated dextran amine (BDA) into the DCN in guinea pigs to label the CN-MG pathway. They observed substantial axonal labeling in the MG. There was much more label contralaterally than ipsilaterally, and the majority of label was located within the MGm. How did they label so many axons with injections into the DCN if, as suggested by the present results, the DCN has relatively few cells that project to the MG? DCN axons may have extensive axonal arbors in the MG. If this is the answer, then a large portion of the CN-MG pathway (the part that originates in the VCN) may have gone unlabeled in the earlier study. An alternative explanation is that many of the axons labeled in the earlier study were from VCN cells that send a collateral to the DCN, where the tracer was deposited. Doucet and Ryugo (2003) described labeling in the lateral superior olivary nucleus in rats after an injection of BDA into the DCN. Those authors concluded that the labeled axons arose from VCN multipolar cells whose axons were labeled via a collateral projection to the DCN injection site. It seems likely that the same event could have occurred in the guinea pig study by Anderson and colleagues. Interestingly, Anderson et al. (2006) tried to activate DCN cells by antidromic stimulation from the MG. They were unable to find such cells, which may reflect the small number of DCN cells involved in the projection. It would be of interest to see if small deposits of BDA into the VCN of guinea pigs would label axons in the MG that differed in morphology or distribution from those labeled by DCN injections. Such data will prove essential to acquiring a complete picture of the CN-MG pathway.

### CELL TYPES

It is hard to speculate on the identity of the DCN cells labeled in the current study. While a very few cells were identifiable as giant or fusiform cells, the majority were located in the deep DCN and appeared to belong to a different cell class. There are a variety of cell types in this layer (Lorente de Nó, 1981; Oertel and Young, 2004), but few are known to have extrinsic projections. A few cells in the deep layer project to the contralateral CN (Cant and Gaston, 1982; Wenthold, 1987; Shore et al., 1992; Schofield and Cant, 1996; Alibardi, 2000). The most common cell type in the deep layer is the vertical cell (also known as tuberculoventral cells or corn cells; Lorente de Nó, 1981; Wickesberg et al., 1991; Kolston et al., 1992; Kuo et al., 2012). These cells are glycinergic and inhibit a variety of other cell types in both the DCN and the VCN (reviewed by Oertel and Golding, 1997; Oertel and Young, 2004). It would be surprising if they project to the MG as there is no evidence for a glycinergic innervation of the thalamus (e.g., Vater et al., 1992; Winer et al., 1995). It is likely that CN-MG cells are members of another cell class. Lorente de Nó (1981) describes other “polymorphic” or “polygonal” cells in the deep DCN; perhaps one of these types contributes to the CN-MG pathway.

The most common type of CN-MG cell is a small multipolar cell in the VCN. Multipolar/stellate cells in the VCN have been divided into several groups (reviewed by Cant and Benson, 2003). We will use the T-stellate/D-stellate terminology developed in mouse. T-stellate cells likely correspond to planar cells, type I multipolar cells, and chopper cells (see discussions in Cant, 1981; Smith and Rhode, 1989; Oertel et al., 1990, 2011; Doucet and Ryugo, 1997, 2006; Cant and Benson, 2003; Doucet et al., 2009). Several features of T-stellate cells suggest that they are the likely contributors to the CN-MG pathway. Multipolar cells that project to the IC are of the T-stellate variety. Our results with double-retrograde tracing indicate that some VCN cells project to both the IC and the MG; we conclude that these are T-stellate cells. T-stellate cells also send an axon collateral to the DCN, consistent with the discussion above that these cells could have contributed to the axons labeled by anterograde transport from DCN. The other major class of multipolar cell is the D-stellate/radiate neuron. These neurons have radiating dendrites and broad frequency tuning. They project to the DCN and the contralateral CN and are thought to inhibit their targets via glycinergic transmission (Wenthold, 1987; Alibardi, 1998; Doucet et al., 1999, 2009; Needham and Paolini, 2003; Shore et al., 2003). The available evidence suggests that D-stellate cells do not project to the MG. Consistent with this conclusion is a preliminary observation (based on observations in two animals) that no CN cells are double-labeled after injection of different retrograde tracers into the contralateral CN and the contralateral MG (unpublished results).

As Cant and Morest (1984) noted, “small cells” of the VCN comprise a heterogeneous group. Some of them may not belong to either the T-stellate or D-stellate classes. As shown in **Figures 3** and **4**, many CN-MG cells are located near the margins of the VCN, and may be associated with the small cell cap. These areas have been associated with type II cochlear nerve input, and may play an important role in intensity coding (discussed below).

### FUNCTIONAL IMPLICATIONS

Perhaps the most obvious implication of a direct projection from the CN to the MG, one that bypasses the myriad of other auditory nuclei in the superior olive, lateral lemniscus and IC, is to provide a fast signal to the auditory thalamus. This supposition is supported by physiological studies demonstrating that cells in the MGm have the shortest latency of all MG cells in response to acoustic stimuli (Anderson and Linden, 2011). Such a signal may serve to “prepare” the target area for further processing related to that stimulus. Additional insights into function may be gained by considering both the cells of origin and the MG subdivisions targeted by the pathway. Previous discussions have focused on the DCN as the source of the pathway and the MGm as the target. Our results suggest that the VCN is the predominant source of projections (at least in guinea pigs) and that the functional views should be expanded accordingly. In addition, we discovered collateral projections to the MG and IC, which imply that some functions are likely to involve both the IC and the MG. Finally, there remain questions about the targets of the pathway in the MG.

What implications arise from the contributions of VCN cells to the CN-MG pathway? T-stellate cells have narrow dendritic fields and correspondingly narrow frequency tuning (Oertel et al., 2011), so projections from T-stellate cells could provide the MG with finely tuned information about acoustic inputs. T-stellate cells also encode stimulus envelope, and as such may play an important role in understanding complex sounds such as speech (reviewed by Oertel et al., 2011). Small cells, in the small cell cap or around the margins of the VCN, have also been identified as projecting to the MG in rats (Malmierca et al., 2002) and guinea pigs (present study). Cells in this area have been associated with high dynamic range with respect to stimulus intensity (Ghoshal and Kim, 1996). These cells have been implicated in both intensity coding and in projections to the medial olivocochlear cells that, in turn, modulate the gain of the cochlea (Benson and Brown, 1990; Brown, 2011). As Malmierca et al. (2002) suggested, if these properties also apply to the CN-MG cells, this direct pathway may provide higher levels of the system with information about actual stimulus intensity. Such information may play a role in perception and also in descending systems (e.g., corticofugal pathways) that could modulate the sensitivity of cells at many levels of the auditory pathway.

The discovery of collateral projections to the MG and the IC also carry implications for function. From a technical perspective, multi-labeling with retrograde tracers is an effective method for identifying branched projections, but generally underestimates the number of cells with such projections (Schofield et al., 2007). The limitations can be reduced by employing different combinations of tracers in different experiments, thus avoiding the limitations associated with any particular tracer. To this end, we used multiple combinations of tracers (**Table 1**). We also varied the tracer injected into a given target; e.g., the MG was injected with FluoroGold, green beads, or Fast Blue for the double-label experiments, and the IC was injected with green beads, red beads, FluoroGold or Fast Blue. The use of multiple tracers in different combinations across a large number of animals provides strong evidence for a collateral projection from CN cells to the contralateral IC and contralateral MG. We observed that up to 37% of the VCN cells that were labeled by tracer injected into the MG were also labeled by tracer injected into the IC. In contrast, we found no evidence for CN cells that have collateral projections to several other combinations of targets (e.g., both MGs; ipsilateral IC and either MG; ipsilateral MG and either IC). The absence of collateral projections in these patterns contrasts directly with the presence of collateral projections to the contralateral IC and contralateral MG. Further, the quantitative analysis of the latter pattern suggests that a substantial portion of the MG-projecting CN cells have axon branches that innervate both the MG and the IC. From a functional standpoint, collateral projections suggest that the same information is sent to each target and that the function(s) of each projection may be similar. Thus, the collateral projection to the IC could provide the same fast “preparatory” signal and/or information about intensity coding that was suggested above for the projection to the MG. In any case, the existence of branching projections should be considered as more information is gathered about the physiological properties and likely functions of the CN-MG pathway.

The MGm is clearly the major target of the CN-MG projections. The traditional view of the MGm is that the neurons are relatively non-selective for acoustic stimuli. They are broadly tuned for features such as stimulus frequency, they have a lower response probability and longer latency of responses compared to neurons in the MGv and the lemniscal pathway (Calford and Aitkin, 1983; Rouiller, 1997). A recent investigation in mice suggests that the MGm contains (and may be dominated by) cells that are more “lemniscal” in their response properties than previously believed. Specifically, many MGm cells have a high probability of response, show sharp tuning for stimulus frequency and respond at the shortest latencies of cells across all MG subdivisions (Anderson and Linden, 2011). The short latencies could be explained in part by the direct projections from the CN. The “lemniscal” properties, such as narrow frequency tuning and high response probability, are also compatible with projections from VCN T-stellate cells as well as cells in the deep DCN (Oertel and Young, 2004).

The MGm projects broadly to auditory cortex and also is a major source of subcortical auditory projections to the amygdala (Doron and Ledoux, 1999). This aspect of circuitry has been closely associated with fear conditioning and with associative plasticity in both the thalamus and the auditory cortex (e.g., Weinberger, 2007, 2011). It would appear relevant that the CN-MG pathway provides a fast route for acoustic information to reach the thalamus and, presumably, the auditory cortex and amygdala as well. The discovery of T-stellate projections to the MG combined with the physiological results of Anderson and Linden (2011) suggest that the MGm is receiving more “refined” (i.e., lemniscal) input than previously appreciated. It will be interesting to determine how this fast, specific input to the MGm contributes to plasticity and conditioning.

## AUTHOR CONTRIBUTIONS

Designed research, wrote the paper: Brett R. Schofield; performed research, analyzed data: Brett R. Schofield, Susan D. Motts, Jeffrey G. Mellott, and Nichole L. Foster.

## Conflict of Interest Statement

The authors declare that the research was conducted in the absence of any commercial or financial relationships that could be construed as a potential conflict of interest.

## Abbreviations

7: facial nucleus
8vn: vestibular nerve root
APT: anterior pretectal nucleus
Cb: cerebellum
CN: cochlear nucleus
cnr: cochlear nerve root
d: deep layer of DCN
DCN: dorsal cochlear nucleus
f: fusiform cell layer of DCN
FB: fast blue
FD: fluorescein dextran
FG: FluoroGold
GB: green beads
grca: granule cell area of CN
IC: inferior colliculus
ICc: central nucleus of IC
ICd: dorsal cortex of IC
IClc: lateral cortex of IC
icp: inferior cerebellar peduncle
m: molecular layer of DCN
MG: medial geniculate body
MGd: dorsal division of MG
MGm: medial division of MG
MGsg: suprageniculate division of MG
MGv: ventral division of MG
MVe: medial vestibular nucleus
NBIC: nucleus of the brachium of the IC
py: pyramid
RB: red beads
sp5: spinal trigeminal tract
SpVe: spinal vestibular nucleus
VCN: ventral cochlear nucleus
